# Nodular Mucinosis of the Breast: A Case Report

**DOI:** 10.7759/cureus.66396

**Published:** 2024-08-07

**Authors:** Ana Y Sandoval-Mussi, Ana S Armenta-Quiroga, Alba Mayra Padilla-Correa, Roberto Enrique Hernandez Peña

**Affiliations:** 1 General Surgery, ABC Medical Center, Mexico City, MEX; 2 General Medicine, Universidad Panamericana, Mexico City, MEX; 3 Surgery, ABC Hospital, Santa Fe Campus, Mexico City, MEX; 4 Oncology Surgery, ABC Medical Center, Mexico City, MEX

**Keywords:** nodular mucinosis, rare disease, nodule, breast, mucinosis

## Abstract

This article presents a rare case of nodular mucinosis of the breast, typically manifested as a painless subcutaneous mass in young women. We describe the clinical scenario of a 48-year-old woman who previously underwent benign nodule resection in her 20s at the identical site where nodular mucinosis subsequently developed. This recurrence at the previous resection site underscores the unusual nature of the condition and emphasizes the need for continued vigilance in monitoring patients with a history of such lesions.

## Introduction

Nodular mucinosis of the breast is an uncommon disorder, classified as a stromal lesion. It was initially documented by Wee et al. in 1989 as a nerve sheath myxoma and later described by Michals et al. in 1998 who identified the lesion and stated its typical characteristics [1]. This condition is defined by the presence of myxoid tissue with scattered spindle cells, notably lacking epithelial components. Typically, it manifests in the nipple-areolar region and often presents as a painless, subcutaneous mass [2,3].

In the literature, there have been only 13 reported cases not attributed to trauma in both English and Spanish languages. The majority of these cases have occurred in females, with 10 reported instances in females and three in males [4]. The age range of individuals with reported cases of nodular mucinosis of the breast varies from 15 years, a case reported by Manglik et al. in a supernumerary nipple, to 72 years [4,5]. However, the median age reported by most cases has been seen in young women in the third and fourth decades of life [4].

## Case presentation

A 48-year-old woman with a history of two benign right breast nodule excisions (at ages 23 and 28) and a total hysterectomy at 47 due to primary alveolar soft part sarcoma of the uterine cervix, began her current condition three months prior to seeking medical attention, presenting a nodule in the upper inner quadrant of the right breast, coinciding with the site of the two previously documented lesions. She reported no nipple discharge or changes in coloration but did experience pain when manipulating the area.

**Figure 1:**
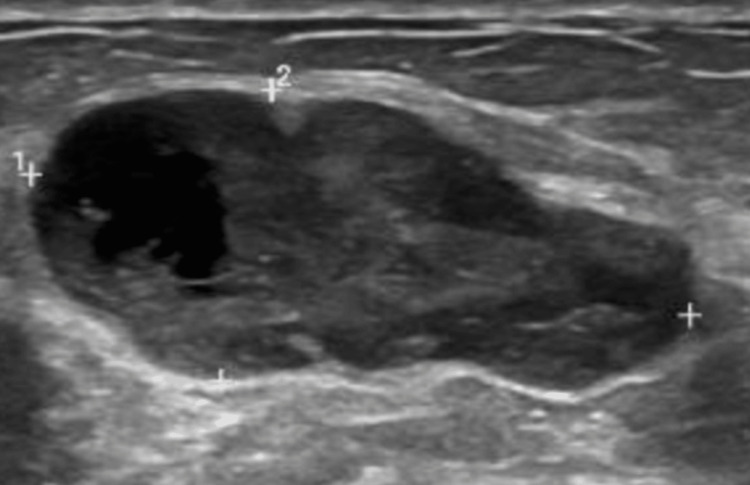
Breast ultrasound of the right breast nodule.

**Figure 2:**
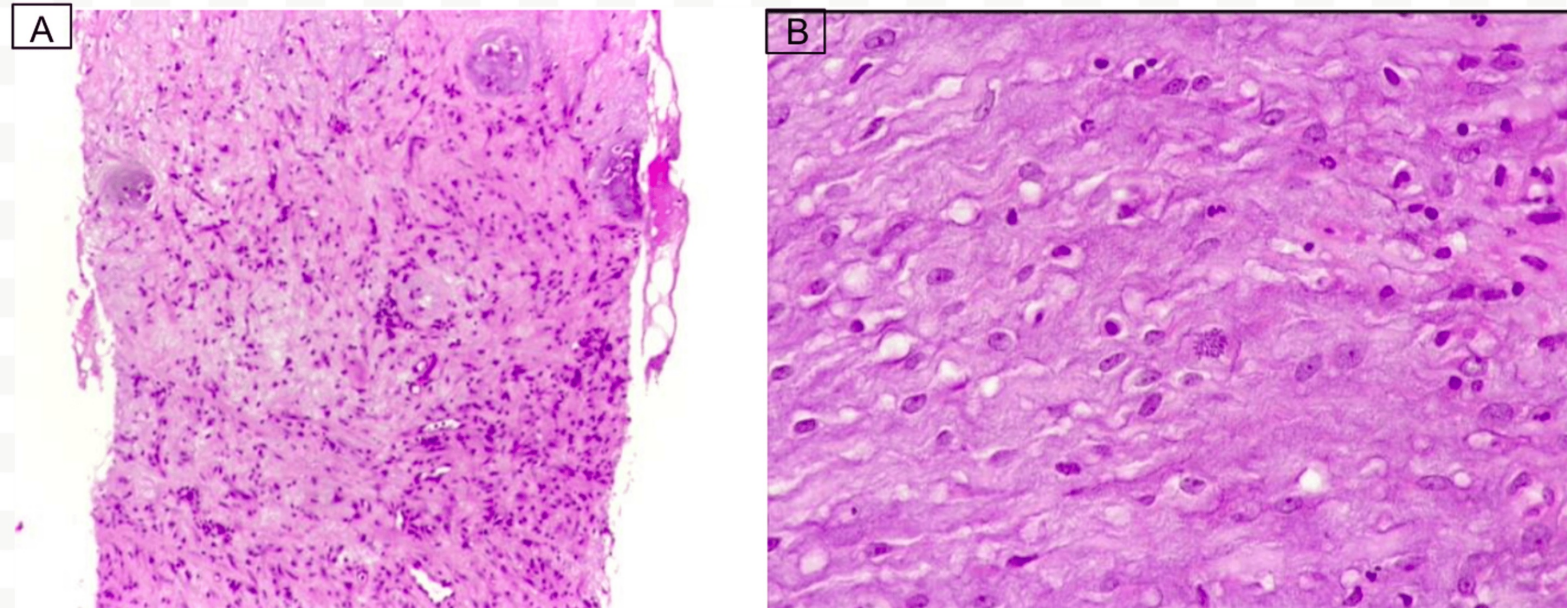
A. Area of the lesion with acute inflammation. B. Spindle cells without atypia, sparse inflammatory cells, loose stroma, and the presence of one mitosis.

Physical examination revealed a well-circumscribed 3 x 3 cm tumor in the upper inner quadrant of the right breast, not fixed to deep planes, without secretions or skin retraction, with no local palpable nodes or changes in the color of the surrounding skin. An ultrasound and biopsy were performed with a nodule in the right breast located between the 2 and 3 o'clock positions, 7 cm from the nipple (Figure 1). Biopsy reported myofibroblastic proliferation with myxoid stroma and acute inflammation, and no areas of necrosis or marked cellular atypia were observed. There are sparse mitoses (one mitosis per 10 fields at 40x). The cells are slightly spindle-shaped. There is no evidence of lymphovascular invasion (Figure 2).

The patient underwent tumor removal via a semilunar incision at the site of the lesion. The procedure involved dissection using monopolar energy until complete resection was achieved.

**Figure 3:**
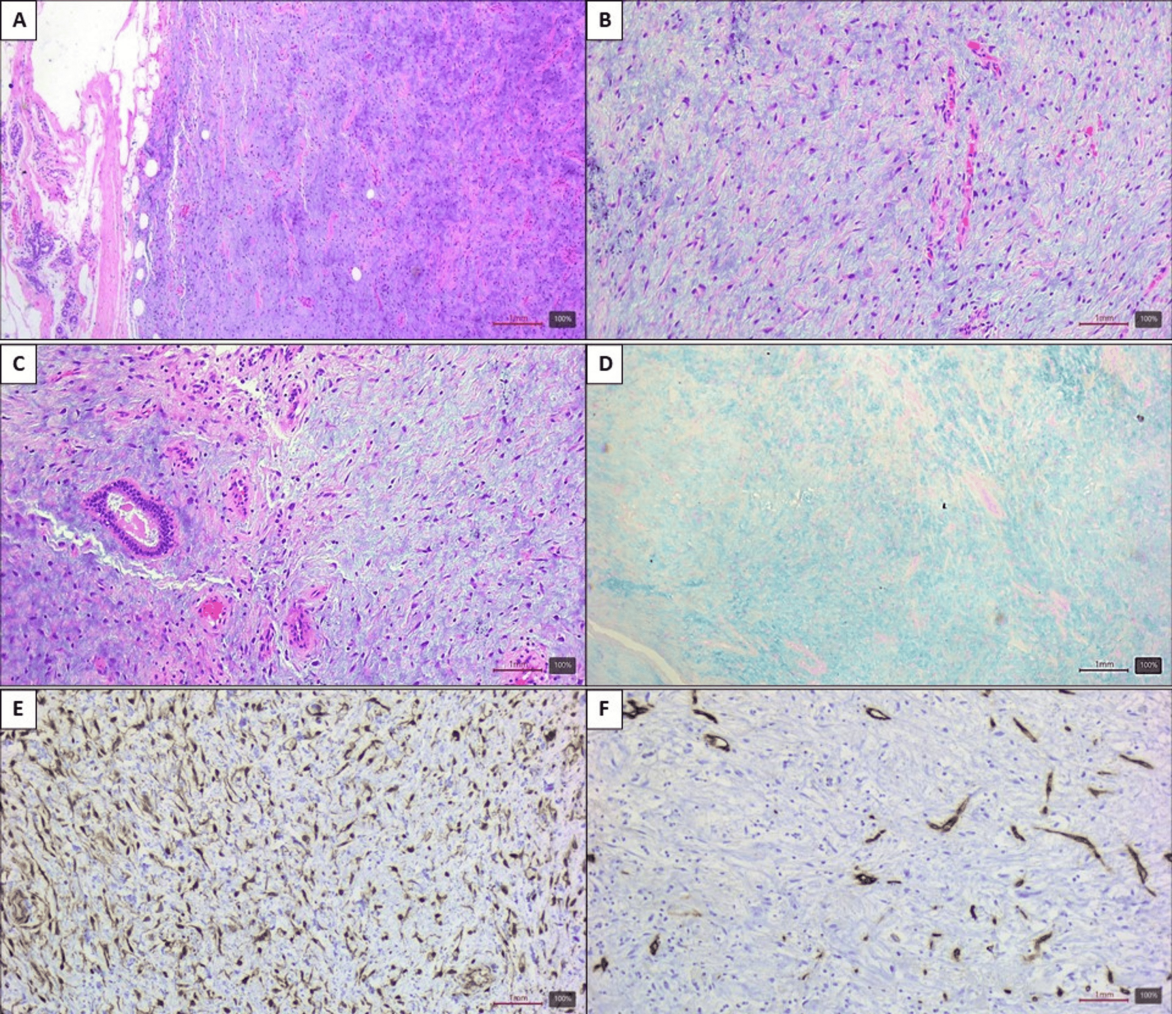
Histopathologic findings. (A) The lesion is a well-circumscribed, not encapsulated solid nodule. (B) The stroma shows a basophilic myxoid substance with scarce bland-looking fibroblasts. (C) Numerous capillaries are found within the lesion and focal dilated mammary ducts. (D) Alcian blue stains the myxoid material in blue. (E) The spindle cells are reactive to β-catenin. (F) The spindle cells show nonreactivity to CD34.

Intraoperative pathologic findings indicated a nodular tissue weighing 10.62 g and measuring 3.9 x 4 x 1.3 cm, with a pale yellow-glistening outer surface. Upon sectioning, the nodule measured 2.6 x 1.5 cm and was well-circumscribed, not encapsulated, and filled with pale tan, gelatinous material, surrounded by white fibrous tissue. Histologically, the lesion displayed myxoid stroma with small spindle cells, bland-looking nuclei, and no atypia or mitosis (Figure 3A, 3B). The proliferation of capillaries and thin-walled vessels was noticed (Figure 3B), as well as focal scarce mammary ducts (Figure 3C). The myxoid stroma was positive for Alcian blue staining (Figure 3D). The spindle cells showed reactivity to β-catenin (Figure 3E) and were negative for CD34 (Figure 3F). These features are consistent with the diagnosis of nodular mucinosis of the breast (Figure 3).

In the immediate postoperative period, the patient was asymptomatic and tolerated oral intake, and the surgical wound showed no signs of complications. Therefore, she was discharged the same day. Follow-up consultations were conducted in the first and third weeks, during which she demonstrated appropriate progress. It was decided to continue doing routine screening with mammography and ultrasound every six months.

## Discussion

Other than the characteristic location, there is no distinctive clinical feature for nodular mucinosis of the breast. It presents as an asymptomatic tumor located in the dermis or subcutaneous cellular tissue. In addition to the clinical features, during the diagnostic approach, ultrasound can be used to appreciate distinctive characteristics. It usually shows a homogenous, hypoechoic mass that is well-circumscribed and lobulated. However, histopathology is essential for diagnosis [4].

Microscopic examination of documented cases reveals distinctive features: a multinodular myxoid proliferation nonencapsulated, yet well-circumscribed, accompanied by scattered spindle cells, occasional histiocytosis, and dilated vascular structures within the mucinous stroma. Staining with Alcian blue and colloidal iron demonstrates positive results for mucin deposition [6].

It is important to differentiate and exclude other benign and malignant conditions. Epithelial cell component mixed in the mucin suggests other mucinous lesions, such as mucinous carcinoma, micropapillary carcinoma in situ with excessive mucin, fibroadenoma with myxomatous stroma, and mucocele-like lesions. Their presence excludes nodular mucinosis of the breast since there are none in the mucin pools [4].

Surgical excision has been chosen as the treatment for the cases described in the literature, and follow-up has shown that there is no recurrence in patients monitored for approximately six months and up to six years. Since it is not linked to any systemic diseases, additional investigations are unnecessary. There has not been any association with the Carney complex in the cases reported in contrast to some of the differential diagnoses, such as myxoid fibroadenomas and mammary myxomas. This is an autosomal dominant syndrome distinguished by multiple neoplasms including myxomas at various sites, endocrine tumors, and lentiginosis related to genetic defects [7].

## Conclusions

Nodular mucinosis of the breast is a rare disease rarely reported in the literature that affects primarily women. It appears with a nonspecific presentation, and it is essential to make a histopathologic diagnosis especially to rule out other lesions. Treatment is exclusively surgical with no need for further interventions due to the characteristics of the disease. There is still much that is not yet known about the pathology such as its origin and if there is a genetic risk that predisposes these individuals to develop this condition.
